# Single cell qPCR reveals that additional *HAND2* and microRNA-1 facilitate the early reprogramming progress of seven-factor-induced human myocytes

**DOI:** 10.1371/journal.pone.0183000

**Published:** 2017-08-10

**Authors:** Emre Bektik, Adrienne Dennis, Prateek Prasanna, Anant Madabhushi, Ji-Dong Fu

**Affiliations:** 1 Department of Medicine, Heart and Vascular Research Center, MetroHealth Campus, Case Western Reserve University, Cleveland, Ohio, United States of America; 2 School of Integrative and Global Majors, University of Tsukuba, Tsukuba, Japan; 3 Department of Biomedical Engineering, Case Western Reserve University, Cleveland, Ohio, United States of America; Murdoch Childrens Research Institute, AUSTRALIA

## Abstract

The direct reprogramming of cardiac fibroblasts into induced cardiomyocyte (CM)-like cells (iCMs) holds great promise in restoring heart function. We previously found that human fibroblasts could be reprogrammed toward CM-like cells by 7 reprogramming factors; however, iCM reprogramming in human fibroblasts is both more difficult and more time-intensive than that in mouse cells. In this study, we investigated if additional reprogramming factors could quantitatively and/or qualitatively improve 7-factor-mediated human iCM reprogramming by single-cell quantitative PCR. We first validated 46 pairs of TaqMan^®^ primers/probes that had sufficient efficiency and sensitivity to detect the significant difference of gene expression between individual H9 human embryonic stem cell (ESC)-differentiated CMs (H9CMs) and human fibroblasts. The expression profile of these 46 genes revealed an improved reprogramming in 12-week iCMs compared to 4-week iCMs reprogrammed by 7 factors, indicating a prolonged stochastic phase during human iCM reprogramming. Although none of additional one reprogramming factor yielded a greater number of iCMs, our single-cell qPCR revealed that additional *HAND2* or microRNA-1 could facilitate the silencing of fibroblast genes and yield a better degree of reprogramming in more reprogrammed iCMs. Noticeably, the more *HAND2* expressed, the higher-level were cardiac genes activated in 7Fs+HAND2-reprogrammed iCMs. In conclusion, *HAND2* and microRNA-1 could help 7 factors to facilitate the early progress of iCM-reprogramming from human fibroblasts. Our study provides valuable information to further optimize a method of direct iCM-reprogramming in human cells.

## Introduction

Heart disease remains the leading cause of death worldwide. In 2013, coronary heart disease alone caused ~1 of every 7 deaths in the United States and led to ~965,000 new or recurrent coronary events [1]. Because cardiomyocytes (CMs) rarely regenerate in adult hearts [2], the loss of CMs typically leads to chronic heart failure. Unfortunately, end-stage heart failure can only be addressed by heart transplantation, which is limited by the number of donor organs available. Recent studies have found that fibroblasts can be directly reprogrammed into chief functional cells in different organs [3–7], including CMs, which holds great promise for regenerative medicine.

Cardiac fibroblasts comprise over half of the cells in the adult heart [8] and thus may provide a large pool of cells from which to generate new CMs through epigenetic reprogramming. We have reported that mouse cardiac and dermal fibroblasts could be directly reprogrammed into induced CM-like cells (iCMs) *in vitro* by a combination of the developmental cardiac transcription factors *Gata4*, *Mef2c*, and *Tbx5* (GMT) [3]. Remarkably, *in vivo* delivery of GMT retrovirus into mouse heart right after myocardial infarction reprogrammed endogenous cardiac fibroblasts into functional iCMs *in situ* and improved cardiac function with attenuated scarring. Other labs around the world have reported success in reprogramming mouse fibroblasts into iCMs with similar cocktails of reprogramming factors [9–13]. Since then, scientists have been enthusiastic to generate more and better reprogrammed mouse iCMs by understanding the mechanism of direct cardiac reprogramming. This has included the use of polycistronic vectors for an optimal stoichiometry of reprogramming factors [14–16], suppression of critical epigenetic barriers [17, 18] and pro-fibrotic signaling [19–21], and optimized culture conditions [22].

Importantly, human cardiac and dermal fibroblasts can also be reprogrammed into cardiac cells [23–25], which is a critical step in translating the technology of cardiac epigenetic reprogramming into a clinical application. Our lab and others have found that the combinations of reprogramming factors used to reprogram iCMs from mouse fibroblasts [3] were not able to reprogram human fibroblasts into iCMs *in vitro*; however, the inclusion of additional reprogramming factors resulted in successful reprogramming. [23–25] In our study, pairing GMT with *ESRRG* and *MESP1* (GMTEM) induced global expression of cardiac genes and shifted the phenotype of human fibroblasts toward the CM-like state; adding two transcription factors of *myocardin* and *ZFPM2* (GMTEMMZ, 7 factors) could further enhance iCM reprogramming in human fibroblasts. Reprogrammed human iCMs were epigenetically stable and could generate Ca^2+^ transients and action potentials [23]. We did not observe spontaneous beating of human iCMs *in vitro*, even 16 weeks after reprogramming, though our analysis of orthologous gene expression demonstrated that, at the global gene-expression level, human iCMs were reprogrammed to a degree similar to mouse iCMs reprogrammed by GMT *in vitro* [23]. Similar observations were also observed in other labs with different combinations of reprogramming factors [24, 25], suggesting that iCM reprogramming is both more difficult and more time-intensive in human fibroblasts. In order to translate this promising approach of iCM reprogramming, therefore, it is important and necessary to understand the mechanism of human iCM reprogramming and optimize the protocol to produce not only a high yield but also a better quality of reprogrammed human iCMs [26].

Here, we advanced the recent development technique of single cell analysis and identified a panel of 46 TaqMan^®^ primers/probes that have high efficiency and sensitivity for single-cell qPCR. We found that the expression profiles of these 46 genes in a single cell were able to distinguish the populations of H9 human embryonic stem cell (ESC)-differentiated CMs (H9CMs) from human fibroblasts, including human dermal fibroblasts (HDFs) and H9 ESC-differentiated fibroblasts (H9Fs). Our single-cell qPCR assays confirmed that human iCM reprogramming was progressing gradually with time, yielding a better reprogramming degree in 12-week reprogrammed iCMs than that in 4-week iCMs. Importantly, we found that none of the additional factors quantitatively enhanced the reprogrammed efficiency, but, compared to GMTEMMZ alone, additional *HAND2* or microRNA-1 could help GMTEMMZ with a facilitated reprogramming progress and yielded more iCMs with a better quality earlier

## Methods

### Human ESCs culture and differentiation

Stable transgenic αMHC-mCherry H9 human ESCs [27] were grown on Matrigel (BD) in mTeSR1 media (StemCell Technologies). H9 ESCs were differentiated into αMHC-mCherry^+^ CMs (H9CMs) by a direct-induction method of cardiomyocytes-differentiation with Chir99021 and IWP4 [28] and were harvested by FACS Aria II (BD Biosciences) 3 weeks post-differentiation. Human ESCs were differentiated into fibroblasts by EB methods and, 3 weeks post-differentiation, αMHC-mCherry^–^/Thy1^+^ H9-differentiated fibroblasts (H9Fs) were purified with APC-conjugated anti-human Thy1 antibody (eBioscience) by FACS Aria II. These purified H9Fs were further cultured and expanded in M106 medium with LSGS (Invitrogen) and H9Fs at passage numbers between 6 and 12 were used for reprogramming.

### Direct cardiac reprogramming with retroviral infection

To generate retroviruses for direct cardiac reprogramming, each pMX retroviral vector of transcription factors, together with retroviral packaging vectors (*gag*/*pol* and VSVG, purchased from Alstem), was transfected into HEK293FT cells using FuGENE 6 (Promega) to generate retroviruses of reprogramming factors. Virus-containing supernatants were pooled and used for transduction of human fibroblasts. The virus-containing medium was replaced with DMEM/M199 medium 24 hours after retroviral infection and changed every 3 days [23]. The reprogramming efficiency of αMHC-mCherry^+^ cells and cardiac troponin-T (cTnT) was analyzed on the LSR-II (BD).

### Single-cell quantitative (q) PCR

Following the protocol of single cell capture and validation [29], Thy1^+^ H9Fs, αMHC-mCherry^+^ H9CMs, and reprogrammed αMHC-mCherry^+^ iCMs were first sorted out by FACS Aria II. With a calcein-AM staining (Live/Dead^®^ cell viability/cytotoxicity kit, Invitrogen), those FACS-purified cells were used for single-cell capture by C1 Single-Cell Auto Prep System (Fluidigm). Every captured well was imaged to confirm a successful capture of a single living cell, followed by reverse transcription and pre-amplification in C1 Single-Cell Auto Prep System. The qPCR assay was performed with these pre-amplified cDNA samples by BioMark^™^ HD System (Fluidigm) to profile gene expression in each individual cell [30]. All TaqMan^®^ primers/probes (Applied Biosystems) used in our study are listed in S1 Table.

### Data analysis of single-cell gene expression profile

The SINGuLAR^™^ Analysis Toolset 3.0 (Fluidigm) was used to analyze all the data of the single-cell qPCR assay. In order to quantitatively evaluate the gene expression level in individual cells, the C_t_ value 30 represents a single copy of a transcript and was subtracted by the C_t_ value of each sample to convert C_t_ values to Log_2_ expression values (Log_2_Ex), which represent transcript levels above background. SINGuLAR defines the outlier threshold as the expression value that is the 15^th^ percentile for those samples, or the value at which 85% of the gene expression values are above that line. Samples whose median expression values are less than the outlier threshold were identified as outliers and excluded from further analysis. To visualize the expression profile, violin plots, which depict the probability density of the data at different values, were generated to compare histograms for multiple genes in each cell population. A hierarchical clustering assay was utilized to assess the progress of 4-week and 12-week reprogrammed iCMs. Individual cells that are more similar to each other were clustered into a group based on their gene expression profiles and were ranked into three subpopulations to quantitatively evaluate the effect of additional factor on the quality of iCM-reprogramming.

A principal component analysis (PCA) and a t-distributed stochastic neighbor embedding (t-SNE) were performed to globally visualize a variance in the data set of single-cell gene-expression profiles. The objective of running a PCA is to reduce dimensionality of a data set and to account for the variation in as few variables as possible, while retaining most of the original variation information. t-SNE minimizes the divergence between the distribution measuring pairwise similarity between the input samples and the one measuring the same between the points in reduced dimensional space [31]. In this specific problem, we reduce the dimensionality to two and demonstrate the optimal separation of samples using the first two components.

### Statistical analyses

Data are expressed as mean ± standard deviation. Differences between groups were examined for statistical significance using Student’s t-test or chi-square test. *P* values of <0.05 were regarded as significant.

## Results

### Validation of TaqMan^®^ primers/probes for single-cell qPCR

**Fig 1:**
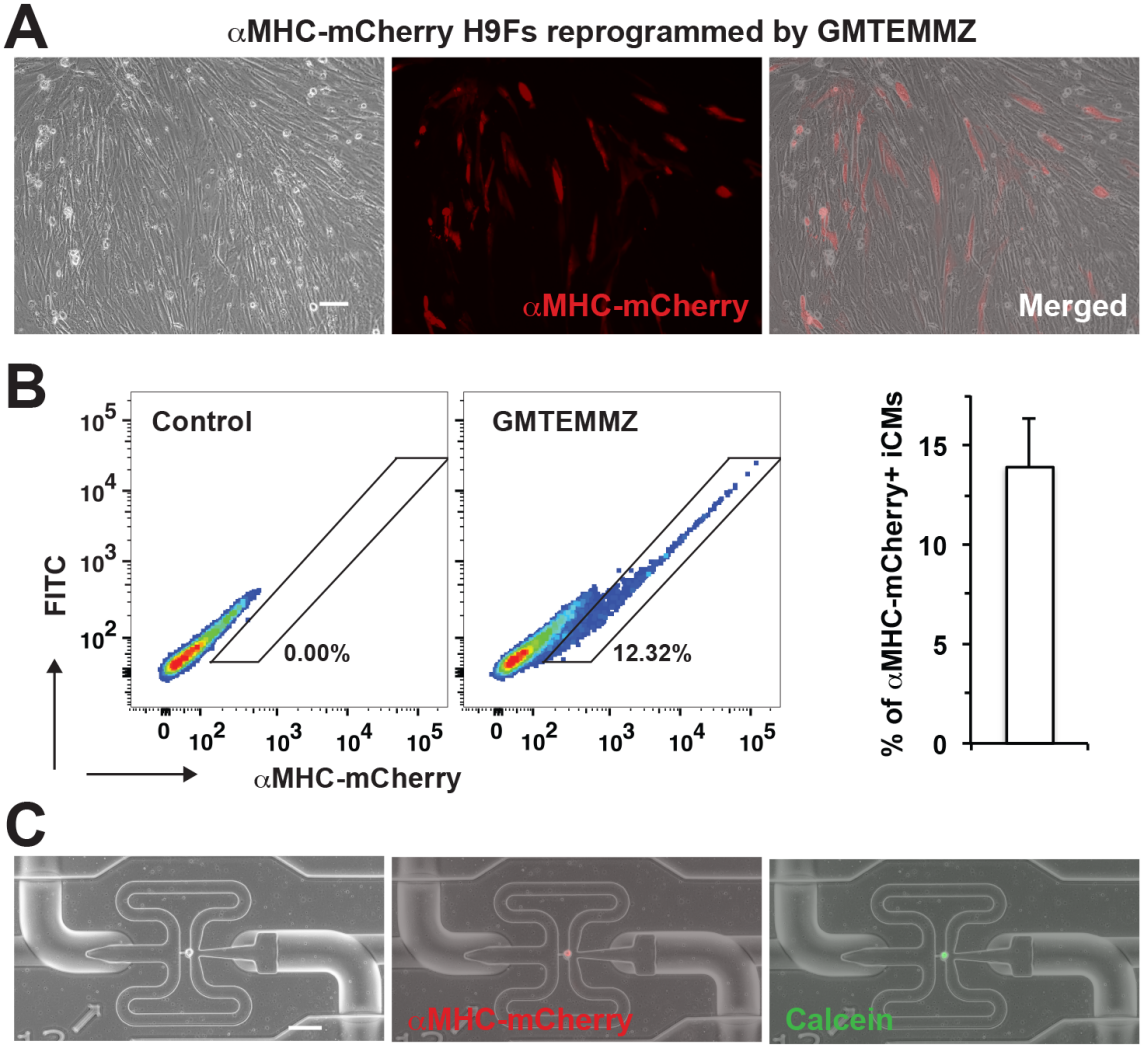
Harvesting individual reprogrammed αMHC-mCherry^+^ H9F iCMs for single-cell qPCR. A) Many αMHC-mCherry^+^ iCMs were observed in reprogrammed H9Fs 4 weeks after retroviral infection of GMTEMMZ. B) αMHC-mCherry^+^ iCMs were quantified and purified by FACS sorter. C) Representative single αMHC-mCherry^+^ iCM captured by C1 Single-Cell Auto Prep System. A positive staining of calcein indicates cell viability. Scale bars, 20 μm.

In addition to the fluorescence reporter αMHC-mCherry, the activation of more cardiac enriched genes and the silence of fibroblast genes in individual cells, not in a pooled population, will estimate the reprogramming process more precisely and evaluate the quality of reprogrammed iCMs [32]. This becomes a feasible approach with the C1 Single-Cell Auto Prep System [29], by which we harvested individual reprogrammed iCMs to profile the gene expression at a single-cell level. Consistent with our previous study [23], retroviral infection of GMTEMMZ efficiently reprogrammed H9Fs into αMHC-mCherry^+^ iCMs (Fig 1A). Four weeks after retroviral infection, αMHC-mCherry^+^ cells (13.9±2.4%, n = 6) were purified by FACS sorter (Fig 1B) and used for single-cell capture by C1 Single-Cell Auto Prep System. All captured cells were imaged and only single mCherry^+^/calcein^+^ cells (Fig 1C) were used in the following qPCR assay, which ensures a good quality single-cell assay with individual, healthy reprogrammed iCMs.

**Fig 2:**
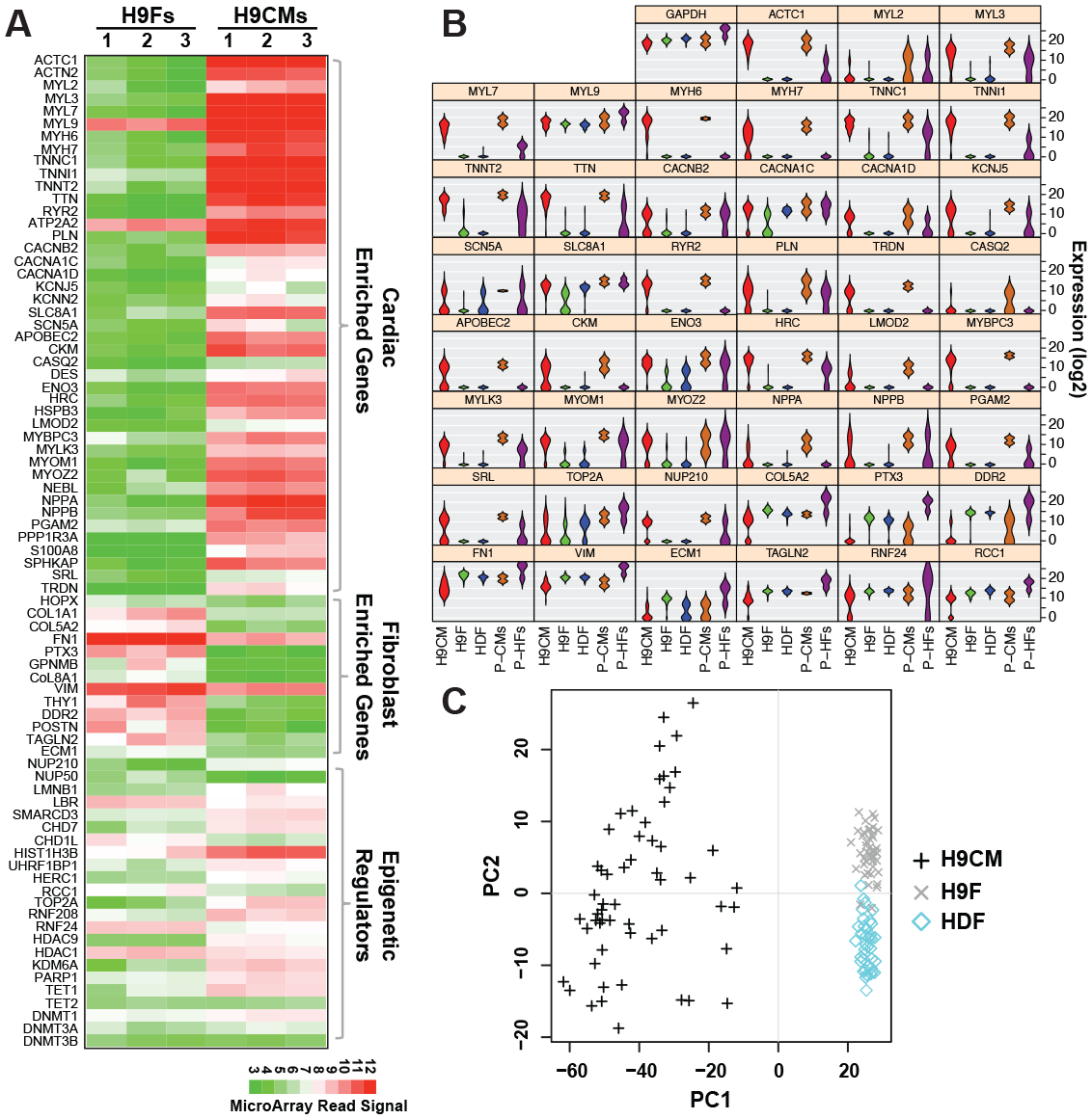
Validation of 96 TaqMan^®^ primers/probes for single-cell qPCR in individual H9CMs (n = 56), H9Fs (n = 45), and HDFs (n = 41). Pooled samples of H9CMs and human fibroblasts (P-CMs and P-HFs) were used as positive controls. A) Heatmap of gene expression profiles in our microarray assay of H9Fs and H9CMs. B) A panel of 46 primers/probes had sufficient efficiency and sensitivity for single-cell qPCR. C) A principal component analysis (PCA) displayed a global view of the expression profile of those 46 genes in individual H9CMs, H9Fs, and HDFs.

In order to achieve a successful single cell qPCR assay, it is important and necessary to validate that each primer/probe works at the single cell level. We carefully analyzed our microarray data and chose 85 genes—9 cardiac transcription factors, 44 cardiac-enriched genes, 12 fibroblast-enriched genes, and 20 epigenetic regulators—which were differentially expressed between H9CMs and H9Fs (Fig 2A). TET2, DNMT3A and DNMT3B were included for a possible comparison with their family genes, and GAPDH was also included as a control. We purchased TaqMan^®^ primers/probes of those 89 genes (S1 Table), which had been validated in regular qPCR and recommended by Applied Biosystems. We also purchased 7 customized TaqMan^®^ primers/probes to detect the 3’- or 5’-UTR region of endogenously-expressed 7 reprogramming factors of GMTEMMZ, but unfortunately those did not work well in single-cell qPCR assay (S1C Fig). To investigate if the primers/probes could efficiently detect the differential expression between CMs and fibroblasts at the single cell level, we successfully harvested 56 samples of individual H9CMs, 45 of individual H9Fs, and 41 of individual HDFs and performed single-cell qPCR. Two cDNA samples of pooled-H9CMs and pooled-H9Fs were included as the positive controls. With a statistical analysis to compare the expression of each gene between fibroblasts and CMS at single-cell level, we found that 10 pairs of primers/probes completely failed in single-cell qPCR assay, although most of them were actually working well with positive controls of pooled samples (S1A Fig). There were 24 pairs of primers/probes that could detect gene expression in individual cells; however, their expressions had no statistical difference between individual H9CMs and human fibroblasts, which is opposite to their expression profiles in pooled samples (S1B Fig). Most primers/probes of cardiac transcription factors were suitable for single-cell qPCR assay (S1C Fig); however, we didn’t include them in follow-up analyses to avoid the potential artifact caused by overexpressed reprogramming factors, which were introduced into fibroblasts by retroviral infection. Therefore, there were 45 pairs of TaqMan^®^ primers/probes that could efficiently detect gene expression and recapitulate the distinctiveness of expression pattern in individual H9CMs and H9Fs (Fig 2B). Together with primers of housekeeping *GAPDH* gene, we performed a PCA assay and could distinguish every single H9CM from human fibroblasts, while individual H9Fs and HDFs had very similar expression profiles (Fig 2C). Noticeably, the expression profiles of these 46 genes also revealed a relatively big variance among individual H9CMs.

### The expression profile of 46 genes could estimate the reprogramming progress of human iCM reprogramming

**Fig 3:**
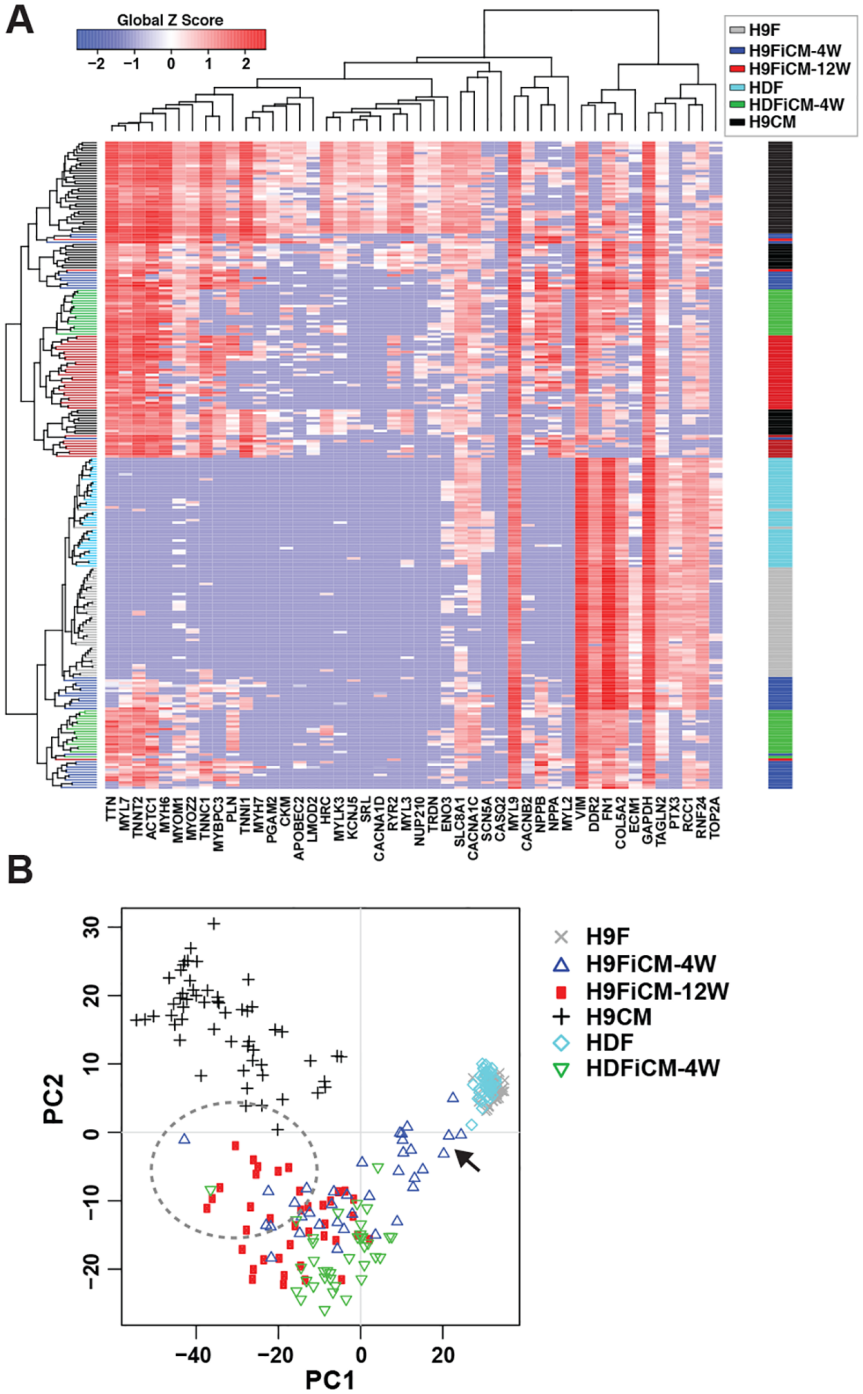
The expression profile of the identified 46 genes is able to estimate the quality of human iCM reprogramming. A) A hierarchical clustering analysis showed that most human iCMs reprogrammed from H9Fs by 7 factors of GMTEMMZ for 12 weeks (H9FiCM-12W, n = 39) were clustered close to H9CMs, while many iCMs reprogrammed from H9Fs (H9FiCM-4W, n = 36) and HDFs (HDFiCM-4W, n = 37) for 4 weeks were clustered close to fibroblasts. B) Principal component (PC) projections of individual cells. The dash-line circle surrounds a population of iCMs that had a good quality of reprogramming; the arrow indicates another population of iCMs that were poorly reprogrammed.

To determine if those 46 primers/probes are sufficient to assess the quality of reprogrammed iCMs, we harvested individual iCMs reprogrammed from H9Fs (H9FiCMs) and HDFs (HDFiCMs) at 4 weeks or 12 weeks post-infection of retroviruses and performed single-cell qPCR along with a hierarchical clustering analysis using the H9CMs and human fibroblasts data. As we expected, all individual H9Fs and HDFs were clustered as two independent populations with a close relationship, while all individual H9CMs were clustered together as a separate population. (Fig 3A). We found that almost all of the iCMs harvested at 12 weeks post-infection (n = 39) were clustered close to H9CMs with many cardiac-enriched genes activated and fibroblast genes silenced; but, around half of 4-week iCMs, including reprogrammed H9FiCMs (n = 36) and HDFiCMs (n = 37), were clustered closer to H9Fs and HDFs (Fig 3A). These results demonstrated that human iCM reprogramming gradually progressed with time, yielding a better degree of reprogramming at 12 weeks than at 4 weeks. A PCA assay provided a better indication that those harvested iCMs had varying degrees of reprogramming. Many 4-week reprogrammed iCMs had a poor degree of reprogramming and were positioned near fibroblasts (indicated by an arrow in Fig 3B); better-reprogrammed iCMs (both 4-week and 12-week) were visualized at a position close to H9CMs (circled region in Fig 3B). Thus, our experiments demonstrated that these 46 primers/probes could be used to estimate the reprogramming degree of individual iCMs.

### Additional cardiac transcription factors didn’t improve the yield of iCMs reprogrammed from human fibroblasts

**Fig 4:**
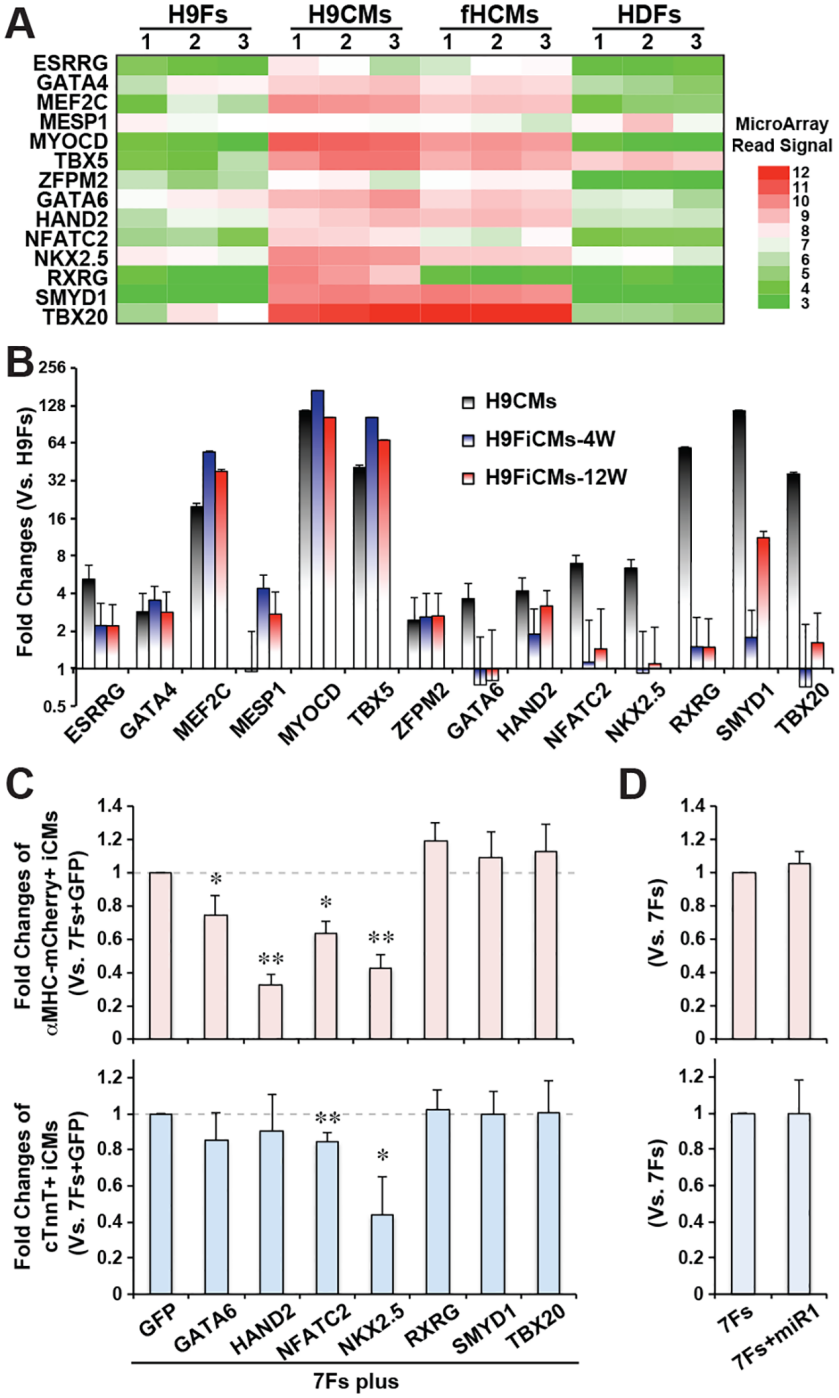
Additional reprogramming factors didn’t increase the yield of αMHC-mCherry^+^ iCMs. A) Heatmap of gene expression profiles for a panel of transcription factors that were expressed at a significantly higher level in pooled CMs (H9CMs and fetal human CMs [fHCMs]) than that in H9Fs and HDFs. B) Fold changes (normalized to H9Fs) of cardiac transcription factors activated in pooled human iCMs reprogrammed by 7 factors (7Fs) of GMTEMMZ for 4 weeks (H9FiCMs-4W) and 12 weeks (H9FiCMs-12W). C-D) The effect of adding one extra transcription factor (C) or microRNA-1 (D) on the induction of αMHC-mCherry^+^ (upper panel, n = 6) or cardiac troponin T (cTnT^+^, lower panel, n = 4) iCMs reprogrammed by 7Fs. *p<0.05, **p<0.01 compared to the control group of 7Fs+GFP.

Since around half of the iCMs reprogrammed by GMTEMMZ for 4 weeks were still at an early stage of reprogramming, we investigated if additional reprogramming factors could enhance or facilitate the progress of direct cardiac reprogramming. In order to choose potentially helpful factors, we re-studied our microarray data and found that, in addition to the seven transcription factors of GMTEMMZ that had been used in our iCM reprogramming, there were some other transcription factors that were expressed significantly higher in H9CMs and human fetal CMs than in H9Fs and HDFs (Fig 4A). Not surprisingly, retrovirus infection upregulated the expression of GMTEMMZ in reprogrammed iCMs to a comparable level of H9CMs (Fig 4B). We noticed that some cardiac-enriched transcription factors were also upregulated to certain levels in reprogrammed iCMs, especially in 12-week reprogrammed cells.

Among those additional transcription factors, *HAND2*, *NKX2*.*5*, and *SMYD1* had been included in our previous study to narrow down the essential combination of reprogramming factors from 19 cardiac transcription factors with a “minus one” strategy [23]. Here, we applied a “GMTEMMZ plus one” to screen potential helpful factors that could further enhance direct cardiac reprogramming. Considering that the presence of one more type of retrovirus might decrease the number of cells infected by all seven retroviruses of GMTEMMZ, we included a control of GFP retrovirus and found that including an additional GFP retrovirus yield a 30.5±0.4% decrease (n = 6, p = 0.006) of αMHC-mCherry^+^ iCMs than that in GMTEMMZ alone group (S2A Fig). Using the GMTEMMZ+GFP group as the control, we found that none of the additional transcription factors could yield a greater number of αMHC-mCherry^+^ or cTnT^+^ iCMs (S2A Fig and Fig 4C); indeed, NFATC2 or NKX2.5 each significantly decreased the yield of both αMHC-mCherry^+^ and cTnT^+^ cells, while HAND2 significantly inhibited the number of reprogrammed αMHC-mCherry^+^ cells. Since microRNA-1 was included in a previous study of human cardiac reprogramming [24], we also investigate if a transfection of microRNA-1 mimic one day after GMTEMMZ-infection could influence the reprogramming efficiency. We found that microRNA-1 had no significant influence on the efficiency of αMHC-mCherry^+^ and cTnT^+^ iCMs reprogrammed by GMTEMMZ (S2B Fig and Fig 4D). Our study demonstrated that iCM reprogramming was not quantitatively enhanced by an extra reprogramming factor.

### HAND2 and microRNA-1 facilitated the early progress of human iCM reprogramming

To investigate if any of these extra factors could improve the quality of reprogrammed iCMs, we harvested individual reprogrammed iCMs at 4 weeks post-infection of retrovirus and performed single-cell qPCR to profile the expression of 46 genes identified above. We directly compared the histogram patterns of each gene among different groups and found that, compared to 4-week iCMs reprogrammed by GMTEMMZ, the introduction of one additional factor significantly decreased the expression of fibroblast-enriched genes, including *COL5A2*, *PTX3*, *FN1*, *VIM*, and *RCC1* (S3 Fig). The expression of Ca^2+^-handling proteins, such as CACNA1D, *CACNB2*, *RYR2*, *PLN* and *CASQ2*, was significantly enhanced by HAND2, SMYD1, or TBX20. HAND2 attenuated the increasing of *MYH6* expression and restrict the expression of *NPPA* and *NPPB* to a similar level of that in H9CMs. The expression of *MYBPC3* was significantly enhanced by HAND2 but suppressed by SMYD1 and TBX20. MicroRNA-1 significantly increased the activation of *RYR2*, *CASQ2*, *MYH6*, *MYH7*, *TNNI1*, and *LMOD2*, but decreased the expression of *MYL9* and *MYOZ2*. Although only with a small number of harvested samples (n = 18), our single-cell qPCR disclosed that RXRG could significantly enhance the activation of *RyR2* and *TTN* and suppress the expression of *COL5A2*, *PTX3*, and *VIM*. Noticeably, some cardiac enriched genes, including *KCNJ5*, *HRC*, and *SRL* (S3 Fig), were not well-activated by any combinations of reprogramming factors, suggesting that this panel of 46 genes might be valuable and useful for further developing an optimized protocol to efficiently generate human iCMs with good quality.

**Fig 5:**
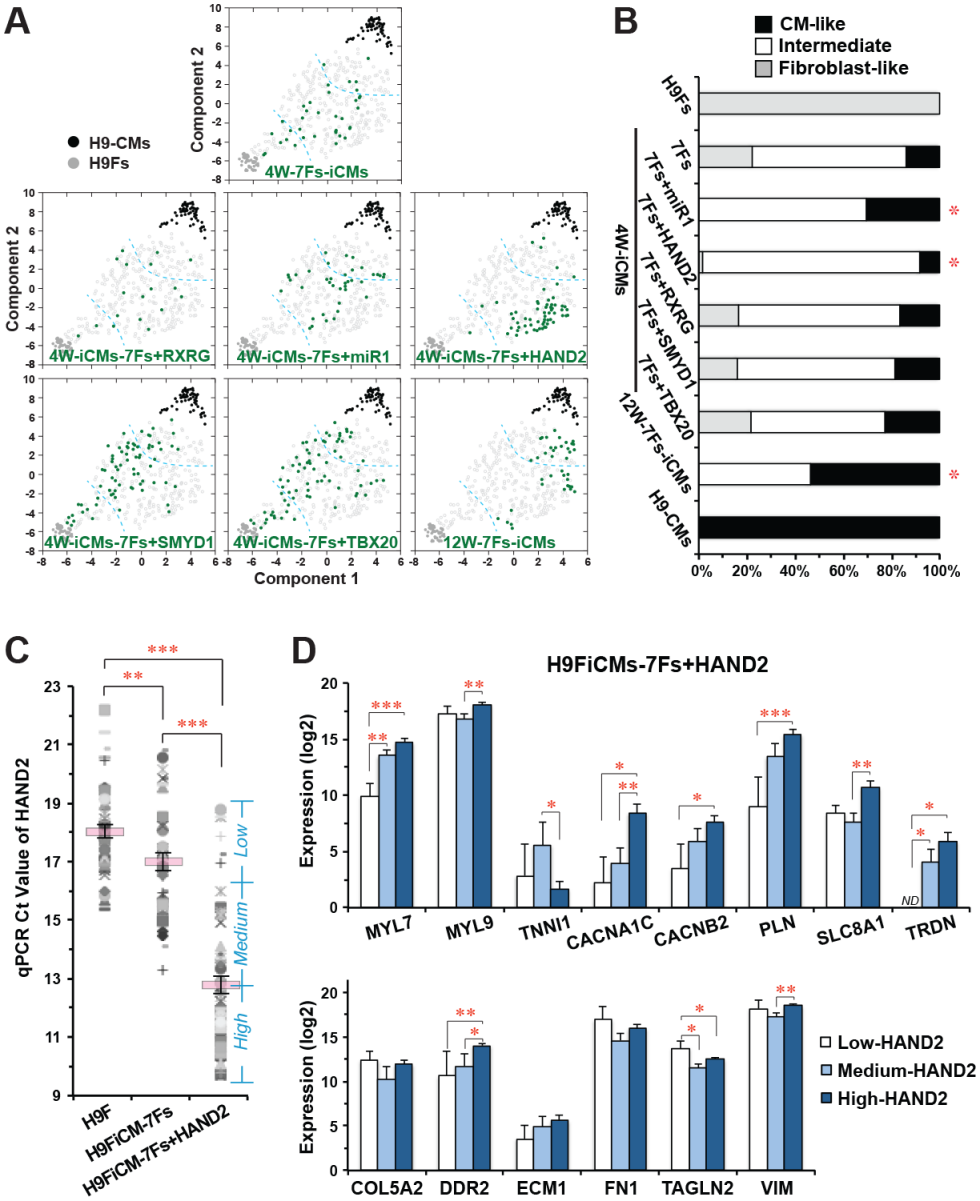
Additional HAND2 or microRNA-1 could facilitate the progress of human iCM-reprogramming by GMTEMMZ 7 factors (7Fs). A) A t-SNE embedding was utilized to visualize the overall reprogramming degree in individual iCMs reprogrammed by 7Fs plus one extra factor, including microRNA-1 (n = 39), *HAND2* (n = 61), *RXRG* (n = 18), *SMYD1* (n = 74), and *TBX20* (n = 74). Two dash lines were inserted to assort iCMs into three populations: fibroblast-like, intermediate-, and CM-like reprogrammed iCMs. The data of H9CMs, H9Fs, 4 weeks-reprogrammed 7Fs-iCMs (4W-7Fs-iCMs) and 12W-7Fs-iCMs were included as control groups. B) The quantification of three subpopulations in panel A showed that additional HAND2 or microRNA-1 significantly decreased the subpopulation of fibroblast-like reprogrammed iCMs, while the CM-like subpopulation was significantly enhanced only in 12W-7Fs-iCMs. *p<0.05 vs. 4W-7Fs-iCMs. C) The expression of HAND2 in individual H9Fs and iCMs reprogrammed by 7Fs or 7Fs+HAND2. 7Fs+HAND2-reprogrammed iCMs were classified into three sub-populations with low-, medium- and high-expression of HAND2. D) Comparison of cardiac (upper panel) and fibroblast-enriched (lower panel) genes among low-HAND2 (n = 5), medium-HAND2 (n = 14) and high-HAND2 (n = 42) subpopulations of 7Fs+HAND2-reprogrammed iCMs. *p<0.05, ** p<0.01, *** p<0.001.

We performed a hierarchical clustering analysis (S4 Fig) and a t-SNE embedding (Fig 5A) and found that both assays could rank the overall reprogramming degree of each individual iCM with a similar result. In order to quantify the influence of additional factors on the reprogramming quality, we assorted those clustered individual iCMs into three sub-populations: some of the iCMs had better quality reprogramming and were embedded at a position closer to H9CMs (termed “CM-like”), a few of them had a poor degree of reprogramming and were embedded closer to H9Fs (termed “fibroblast-like”), and many of them were embedded as a separate population (termed “intermediate”) (Fig 5A). We quantified the percentage of these three sub-populations in each group of iCMs reprogrammed by different combinations of factors and found that 4 weeks after retroviral infection of GMTEMMZ, only 16.7% of reprogrammed cells were CM-like cells, 30.6% of them were still fibroblast-like cells, and the remaining 52.8% iCMs had an intermediate-degree of reprogramming (Fig 5B). However, none of 12-week GMTEMMZ-reprogrammed iCMs were fibroblast-like cells and more than half of them were CM-like reprogrammed iCMs. Most importantly, the fibroblast-like population was significantly decreased in those 4-week iCMs reprogrammed by GMTEMMZ plus HAND2 or microRNA-1 (Fig 5B), while RXRG, SMYD1 and TBX20 had no significant influence on the overall quality of reprogrammed iCMs and the CM-like subpopulation was significantly increased in 12W-7Fs-iCMs.

Adding an additional retrovirus brings a concern how efficiently the additional factor was overexpressed in all reprogrammed iCMs. Our single-cell qPCR assay revealed that *HAND2* was expressed significantly higher in GMTEMMZ-reprogrammed iCMs than that in individual H9Fs, but was still significantly lower than that in GMTEMMZ+HAND2-reprogrammed cells (Fig 5C), suggesting that the additional factor was efficiently overexpressed in reprogrammed iCMs. We noticed a big various expression level of HAND2 and classified GMTEMMZ+HAND2-reprogrammed iCMs into three subpopulations with low, medium and high expression level of HAND2 (Fig 5C) and compared gene-expression profiles among them. We found that many cardiac genes were expressed significantly higher in those iCMs with high-HAND2 than that in iCMs with medium- or low-HAND2 (Fig 5D). Fibroblasts genes were expressed at a similar level among three sub-populations and some of them (i.e. DDR2 and VIM) were even expressed at a higher level in high-HAND2 iCMs, suggesting that the improved silence of fibroblast genes was an overall consequence induced by all reprogramming factors. Our study demonstrated that additional HAND2 and microRNA-1 could help GMTEMMZ to facilitate the early progress of iCM-reprogramming in human cells.

## Discussion

In the current study, we investigated a panel of 96 TaqMan^®^ primers/probes and validated 46 primers/probes that have sufficient efficiency and sensitivity for single-cell qPCR. We found that profiling these 46 genes in individual cells was able to estimate the reprogramming degree of iCMs from human fibroblasts. Importantly, our study reveals that introducing additional *HAND2* or microRNA-1 together with GMTEMMZ could facilitate the early progress of iCM reprogramming with improved activation of cardiac genes and silence of fibroblast genes. Our study provides valuable information to further translate this new technology of epigenetic iCM reprogramming for cardiac regenerative medicine.

Transient overexpression of a combination of defined master regulators can effectively induce cellular reprogramming––converting an alternative phenotype (cardiac muscle cell) from a differentiated cell lineage (fibroblast). Direct reprogramming of iCMs from fibroblasts *in vitro* [3, 9, 12, 13] and *in vivo* [11, 14, 33, 34] opens a potentially new therapeutic avenue to convert resident support cells––cardiac fibroblasts––into iCMs *in situ* and replace lost tissue in a damaged heart [35]. However, other groups as well as ours have found that iCM reprogramming from human fibroblasts is much more difficult than from mouse cells and requires additional cardiac-enriched transcription factors and/or microRNAs [23–25]. Good quality human iCMs were rare and only observed after long-term reprogramming, which was also revealed in 12W-reprogrammed iCMs by our single-cell qPCR. All of these studies demonstrate that more effort is required to further optimize the method of epigenetic iCM reprogramming from human fibroblasts.

At the initial stage of ours and other studies, the activation of αMHC-derived fluorescence reporter and/or one cardiac gene had been utilized to identify a combination of reprogramming factors that are sufficient to convert human fibroblasts into CM-like cells. The strategy of serially removing individual factors from the pool of reprogramming factors is useful and practical to narrow down the most necessary combination of reprogramming factors. However, this strategy might potentially identify a combination of transcription factors that preferentially activate the specific promoter (i.e. αMHC or cTnT) and might not comprise the most optimal combination for cardiac cell fate conversion [36]. Protze et al. [10] measured the activation of a panel of five cardiac genes—*Myh6*, *Myl2*, *Actc1*, *Nkx2*.*5*, and *Scn5a*—in pooled mouse embryonic fibroblasts and investigated the reprogramming consequence of different triplet-combinations of 10 critical transcription factors. Similar, Christoforou et al. [13] studied the activation of 34 endogenous cardiac–enriched genes and evaluated the capacity of cellular reprogramming in pooled reprogrammed mouse fibroblasts, finding that *myocardin* and *SRF* could enhance cardiac reprogramming of GMT. Noticeably, the activation of multiple genes in pooled samples doesn’t really mean that all upregulated genes are activated in the same individual cell and it is not direct evidence to estimate the quality of reprogrammed iCMs. A new developed single-cell qPCR of elastomer-based integrated fluidic circuit allows us to capture individual cells and study gene-expression profiles at the single cell level [30].

The quality and precision of a qPCR assay depends on the efficiency and sensitivity of the primers used. Although all TaqMan^®^ primers/probes used in our study have been validated by regular qPCR with cDNA samples from pooled cells, 10 pairs of primers/probes have low efficiency and failed to detect the expression of target gene at the single cell level, and 24 pairs were not sensitive enough to differentiate the expression profiles between human CMs and fibroblasts. Our validation of single-cell qPCR experiments identified 46 pairs of primers/probes that have sufficient efficiency and sensitivity to detect the expression level of those 46 genes in individual H9CMs, H9Fs, and HDFs. Similar to previous studies [30, 37], our single-cell qPCR assay revealed a diversity of H9CMs (Fig 2C), which might represent H9CMs at different stages of differentiation. Most importantly, the profile of those 46 genes also disclosed the diverse activation level of cardiac genes in human iCMs and could allow us to estimate the reprogramming degree in each individual iCMs (Fig 3). During reprogramming of induced pluripotent stem cells (iPSCs), the profile of 48 validated pluripotent genes in individual cells of reprogrammed fibroblasts revealed that iPSC reprogramming proceeds in two major phases: a prolonged “stochastic” phase followed by a late ‘hierarchical” phase [38]. The profile of our verified 46 genes in reprogrammed individual iCMs revealed that most human iCMs reprogrammed 12 weeks had better quality of reprogramming than 4-week reprogrammed iCMs and suggested that human iCM reprogramming might also have a protracted stochastic phase during which both silence of fibroblast genes and activation of cardiac enriched genes are randomly and gradually processed along the time of programming.

With a “GMTEMMZ plus one” strategy, our single-cell qPCR assay of reprogrammed human iCM disclosed that an additional reprogramming factor helps GMTEMMZ to activate specific cardiac gene(s) (S3 Fig); more importantly, the expression profile of those 46 genes could estimate the overall quality of reprogramming in individual iCMs and revealed that additional *HAND2* or microRNA-1 could facilitate the early progress of iCM reprogramming, indicated by a decreased subpopulation of fibroblast-like iCMs with decreased expression of fibroblast genes and enhanced activation of cardiac genes. Our single-cell qPCR assay suggests that the decreased expression of fibroblast genes is likely an overall consequence induced by all reprogramming factors; importantly, we observed that the more *HAND2* expressed, the higher-level were cardiac genes activated in those 7Fs+HAND2-reprogrammed iCMs, indicating that the higher expression level of *HAND2* direct contribute to the enhanced activation of cardiac genes. Noticeably, we didn’t observe that an additional reprogramming factor could help GMTEMMZ to yield more αMHC-GFP^+^ or cTnT^+^ iCMs (Fig 4C), which is consistent with our previous finding with a “minus one” strategy [23]. These results suggest that both a high yield and a good quality of reprogrammed iCMs should be considered carefully to optimize a proper combination of reprogramming factors.

One limitation of our current study is that an additional factor was delivered by one extra retrovirus, although the significantly higher expression of *HAND2* in GMTEMMZ+HAND2-iCMs indicates an efficient gene-delivery by the extra retrovirus. An approach of linking the gene with a fluorescent reporter by 2A peptide or IRES will be used in future study to better identify cells that express the additional factor.

In summary, considering the success of qPCR assay depends on the quality of the primers, our study has great scientific significance for validating a panel of 46 primer/probe pairs that are adequate in terms of sensitivity and specificity for single cell study. Our rigorous single cell transcript analysis of the reprogramming process in human cells provides valuable information to facilitate the progress of iCM reprogramming in human cells and to further advance the translation of direct cardiac reprogramming for cardiac regenerative medicine.

## Supporting information

S1 Fig Validation of 96 TaqMan^®^ primers/probes for single-cell qPCR in individual H9CMs (n = 56), H9Fs (n = 45), and HDFs (n = 41). Pooled samples of H9CMs and human fibroblasts (P-CMs and P-HFs) were used as positive controls. A) Violin plots of single-cell qPCR showed that 10 pairs of primers/probes failed to detect gene expression at the single cell level. B) 24 pairs of primers/probes failed to distinguish the differential expression profile between H9CMs and human fibroblasts. C) Violin plots of single-cell qPCR with primers/probes of cardiac transcription factors and their 3’ or 5’UTR.
(TIF)

S2 Fig The effect of adding one extra transcription factor or microRNA-1 on human iCM-reprogramming. A-B) Representative FACS plots showing the effect of additional factors on the induction of αMHC-mCherry^+^ (A) or cardiac troponin T^+^ (cTnT^+^, B) iCMs reprogrammed by 7Fs. C) Representative FACS plots showing the effect of microRNA-1 (miR1) on the induction of αMHC-mCherry^+^ (upper panel) or cTnT^+^ (lower panel) iCMs reprogrammed by 7Fs.
(TIF)

S3 Fig Gene expression profiles of reprogrammed iCMs. Violin plots of single cell qPCR showed the expression of the 46 identified genes in the populations of iCMs reprogrammed by 7 factors (7Fs) GMTEMMZ plus one extra factor, including *HAND2*, *RXRG*, *SMYD1*, *TBX20*, and microRNA-1. H9CMs, H9Fs, 4-week, and 12-week GMTEMMZ-reprogrammed iCMs were included as control groups.
(TIF)

S4 Fig Estimating the quality of reprogramming in individual iCMs. A hierarchical clustering assay were performed to evaluate the reprogramming degree of individual iCMs reprogrammed by 7 factors (7Fs) of GMTEMMZ plus one extra factor, including *HAND2* (n = 61), *RXRG* (n = 18), *SMYD1* (n = 74), *TBX20* (n = 74), and microRNA-1 (n = 39). The data of H9CMs, H9Fs, 4-week, and 12-week GMTEMMZ-reprogrammed iCMs were included as control groups.
(TIF)

S1 Table TaqMan® primers used in single-cell qPCR. (PDF)
