# Supplementary figures and images for: Single cell qPCR reveals that additional HAND2 and microRNA-1 facilitate the early reprogramming progress of seven-factor-induced human myocytes
Source: PLoS One. 2017 Aug 10;12(8):e0183000. doi: 10.1371/journal.pone.0183000 (PMC5552090; doi:10.1371/journal.pone.0183000)

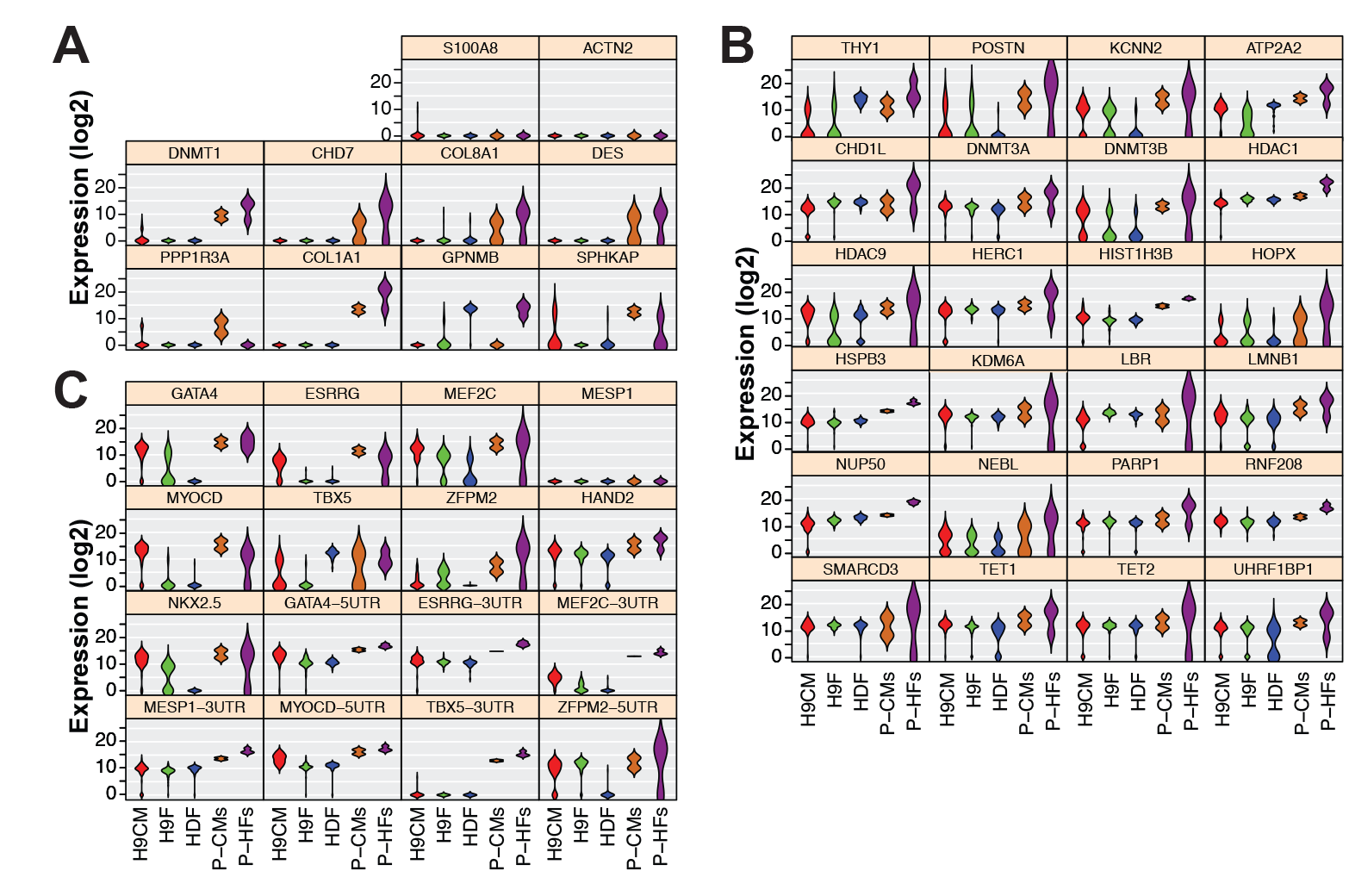

Supplement: S1 Fig — Pooled samples of H9CMs and human fibroblasts (P-CMs and P-HFs) were used as positive controls. A) Violin plots of single-cell qPCR showed that 10 pairs of primers/probes failed to detect gene expression at the single cell level. B) 24 pairs of primers/probes failed to distinguish the differential expression profile between H9CMs and human fibroblasts. C) Violin plots of single-cell qPCR with primers/probes of cardiac transcription factors and their 3’ or 5’UTR. (TIF) [file pone.0183000.s001.tif]

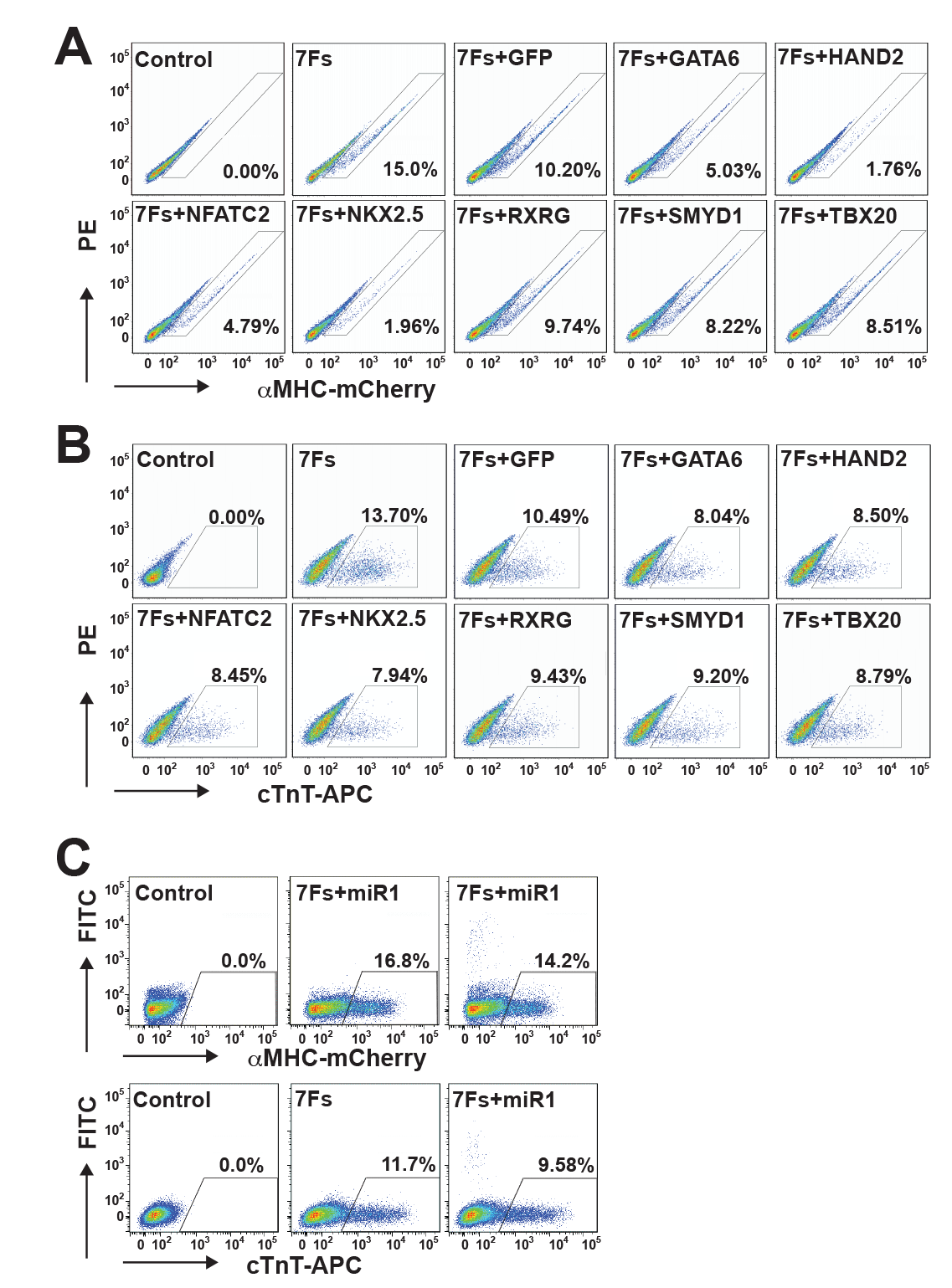

Supplement: S2 Fig — A-B) Representative FACS plots showing the effect of additional factors on the induction of αMHC-mCherry+ (A) or cardiac troponin T+ (cTnT+, B) iCMs reprogrammed by 7Fs. C) Representative FACS plots showing the effect of microRNA-1 (miR1) on the induction of αMHC-mCherry+ (upper panel) or cTnT+ (lower panel) iCMs reprogrammed by 7Fs. (TIF) [file pone.0183000.s002.tif]

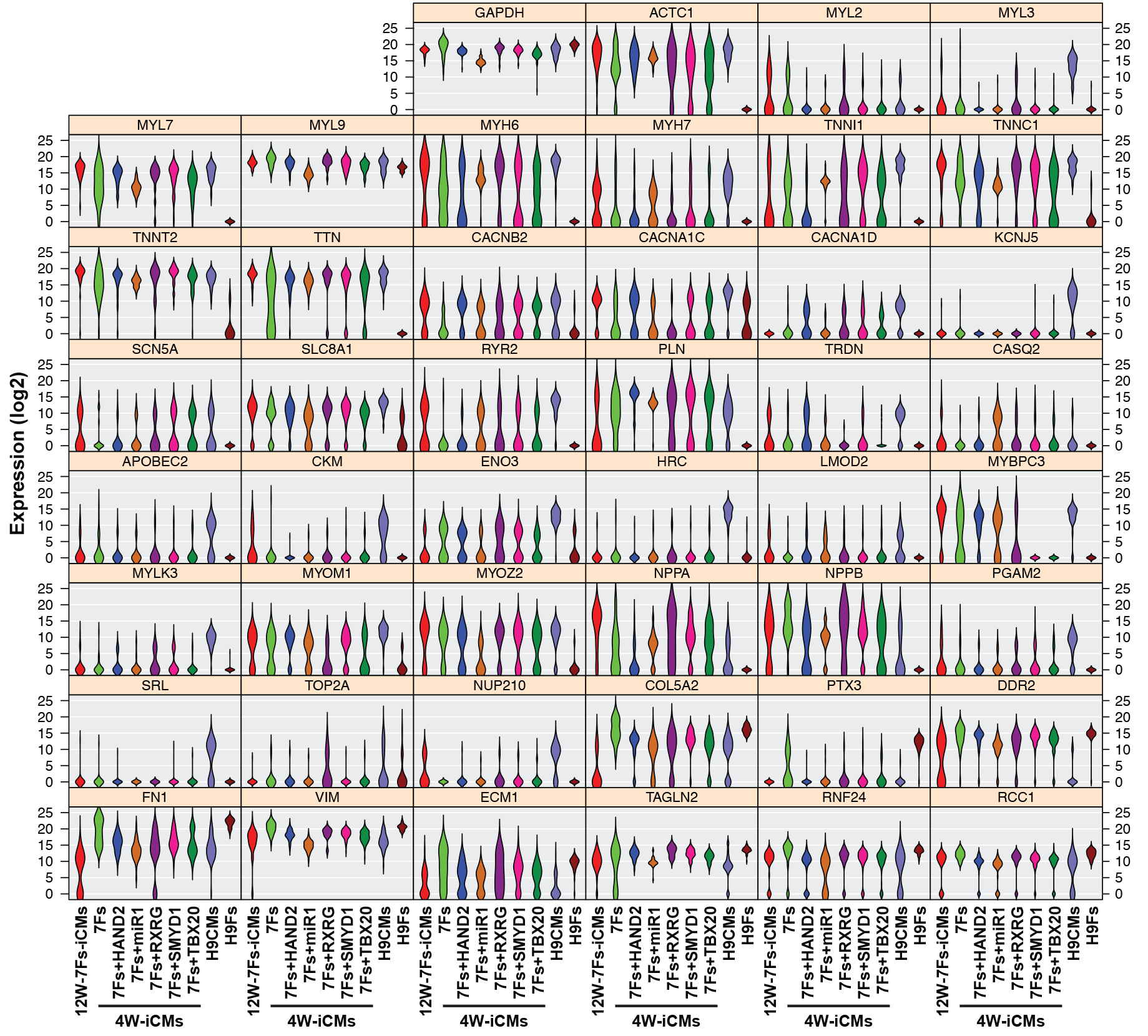

Supplement: S3 Fig — Violin plots of single cell qPCR showed the expression of the 46 identified genes in the populations of iCMs reprogrammed by 7 factors (7Fs) GMTEMMZ plus one extra factor, including HAND2, RXRG, SMYD1, TBX20, and microRNA-1. H9CMs, H9Fs, 4-week, and 12-week GMTEMMZ-reprogrammed iCMs were included as control groups. (TIF) [file pone.0183000.s003.tif]

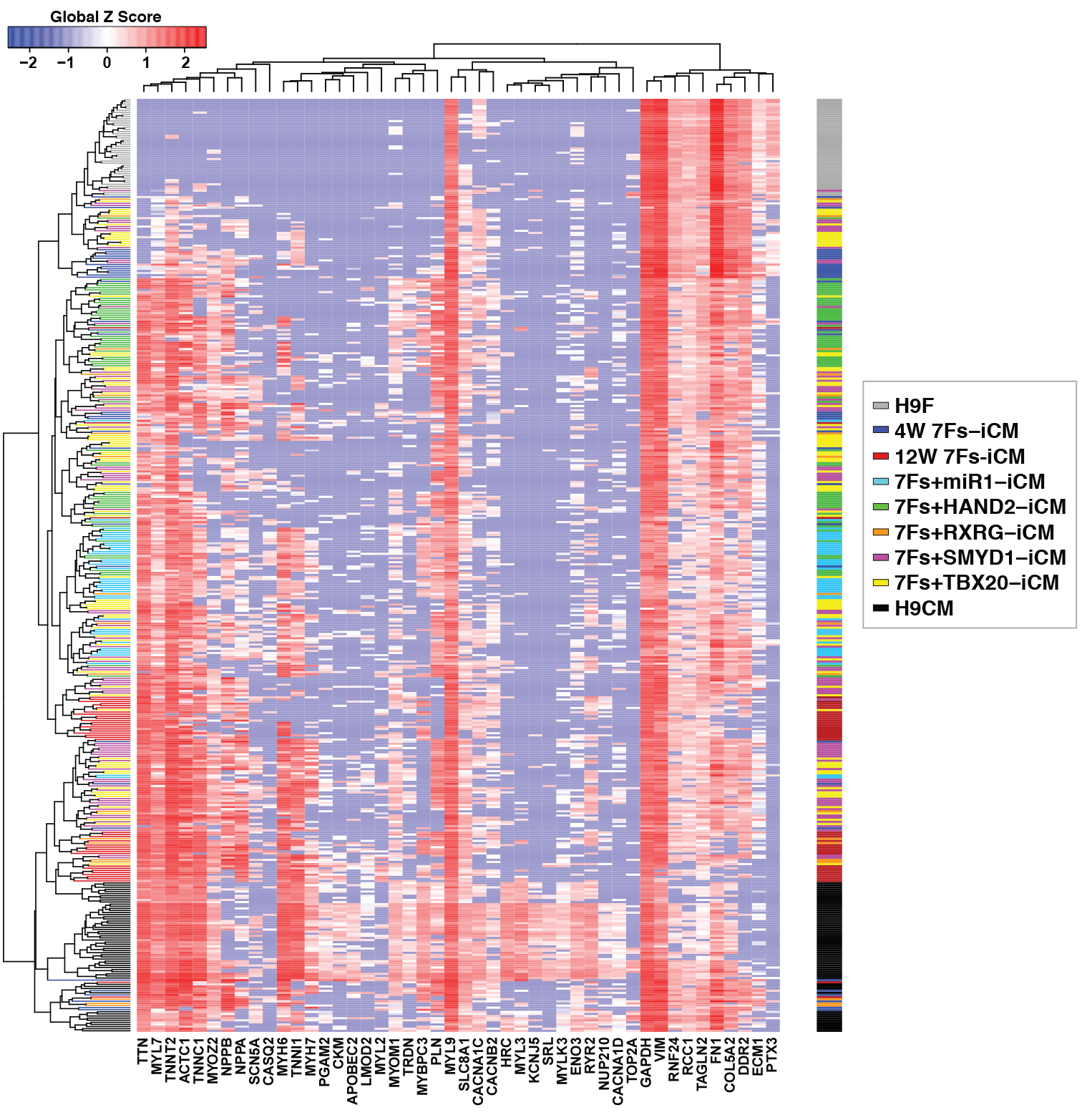

Supplement: S4 Fig — A hierarchical clustering assay were performed to evaluate the reprogramming degree of individual iCMs reprogrammed by 7 factors (7Fs) of GMTEMMZ plus one extra factor, including HAND2 (n = 61), RXRG (n = 18), SMYD1 (n = 74), TBX20 (n = 74), and microRNA-1 (n = 39). The data of H9CMs, H9Fs, 4-week, and 12-week GMTEMMZ-reprogrammed iCMs were included as control groups. (TIF) [file pone.0183000.s004.tif]
