# Supplementary material for: Single cell qPCR reveals that additional HAND2 and microRNA-1 facilitate the early reprogramming progress of seven-factor-induced human myocytes
Source: PLoS One. 2017 Aug 10;12(8):e0183000. doi: 10.1371/journal.pone.0183000 (PMC5552090; doi:10.1371/journal.pone.0183000)
Supplement: S1 Table — (PDF) [file pone.0183000.s005.pdf]

S1 Table. TaqMan® primers used in single-cell qPCR

	Gene Name	Cat.# of TaqMan Primer	Gene Name	Cat.# of TaqMan Primer
Reprogramming Transcription Factors	ESRRG	Hs00155006_m1	ESRRG-3'UTR	
	GATA4	Hs00171403_m1	GATA4-5'UTR	
	MEF2C	Hs00231149_m1	MEF2C-3'UTR	
	MESP1	Hs00251489_m1	MESP1-3'UTR	
	MYOCD	Hs00538071_m1	MYOCD-5'UTR	
	TBX5	Hs00361155_m1	TBX5-3'UTR	
	ZFPM2	Hs00201397_m1	ZFPM2-5'UTR	
	HAND2	Hs00232769_m1	NKX2.5	Hs00231763_m1
Cardiac Enriched Genes	ACTC1	Hs01109515_m1	APOBEC2	Hs00199012_m1
	ACTN2	Hs00153809_m1	CKM	Hs00176490_m1
	MYL2	Hs00166405_m1	CASQ2	Hs00154286_m1
	MYl3	Hs00264820_m1	DES	Hs00157258_m1
	MYL7	Hs00221909_m1	ENO3	Hs01093275_m1
	MYL9	Hs00697086_m1	HRC	Hs01011555_g1
	MYH6	Hs00411908_m1	HSPB3	Hs00272204_s1
	MYH7	Hs01110632_m1	LMOD2	Hs00411574_m1
	TNNC1	Hs00896999_g1	MYBPC3	Hs00165232_m1
	TNNI1	Hs00913333_m1	MYLK3	Hs00294850_m1
	TNNT2	Hs00165960_m1	MYOM1	Hs00187003_m1
	TTN	Hs00399225_m1	MYOZ2	Hs00213216_m1
	RYR2	Hs00892883_m1	NEBL	Hs01590549_m1
	ATP2A2	Hs01566028_g1	NPPA	Hs00383230_g1
	PLN	Hs00160179_m1	NPPB	Hs00173590_m1
	CACNB2	Hs00167861_m1	PGAM2	Hs00165474_m1
	CACNA1C	Hs00167681_m1	PPP1R3A	Hs00366043_m1
	CACNA1D	Hs01073321_m1	S100A8	Hs00374264_g1
	KCNJ5	Hs00942581_m1	SPHKAP	Hs00286757_m1
	KCNN2	Hs01030641_m1	SRL	Hs00328092_m1
	SLC8A1	Hs01062258_m1	TRDN	Hs00196009_m1
	SCN5A	Hs00165693_m1	HOPX	Hs00261238_m1
Fibroblast Enriched Genes	COL1A1	Hs00164004_m1	VIM	Hs00185584_m1
	COL5A2	Hs00893878_m1	Thy1	Hs00174816_m1
	FN1	Hs00365052_m1	DDR2	Hs01025953_m1
	PTX3	Hs00173615_m1	POSTN	Hs00170815_m1
	GPNMB	Hs01095679_m1	TAGLN2	Hs00761239_s1
	COL8A1	Hs00156669_m1	ECM1	Hs00189435_m1
Epigenetic Regulators	NUP210	Hs00227779_m1	RNF208	Hs00846452_s1
	NUP50	Hs00855432_g1	RNF24	Hs00939205_m1
	LMNB1	Hs01059210_m1	HDAC9	Hs00206843_m1
	LBR	Hs01032700_m1	HDAC1	Hs02621185_s1
	SMARCD3	Hs00162003_m1	KDM6A	Hs00958902_m1
	CHD7	Hs00215010_m1	TET1	Hs00286756_m1
	CHD1L	Hs00610997_m1	TET2	Hs00325999_m1
	HIST1H3B	Hs00605810_s1	PARP1	Hs00242302_m1
	UHRF1BP1	Hs00766001_m1	DNMT1	Hs00154749_m1
	HERC1	Hs01032528_m1	DNMT3a	Hs01027166_m1
	RCC1	Hs00154399_m1	DNMT3b	Hs00171876_m1
	TOP2A	Hs00172214_m1		
**Housekeeping gene**			GAPDH	Hs02758991_g1
